# Changes in the Microbiome of Vaginal Fluid after Menopause in Korean Women

**DOI:** 10.4014/jmb.2106.06022

**Published:** 2021-09-03

**Authors:** Sukyung Kim, Hoonhee Seo, MD Abdur Rahim, Saebim Lee, Yun-Sook Kim, Ho-Yeon Song

**Affiliations:** 1 Probiotics Microbiome Convergence Center, Soonchunhyang University, Asan, Chungnam 31538, Republic of Korea; 2 Department of Microbiology and Immunology, School of Medicine, Soonchunhyang University, Cheonan, Chungnam 31151, Republic of Korea; 3 Department of Obstetrics and Gynecology, Soonchunhyang University Cheonan Hospital, Chungnam 31151, Republic of Korea

**Keywords:** Vaginal microflora, premenopausal, postmenopausal, metagenomics, Korean women

## Abstract

Various microorganisms reside in the human vagina; the vaginal microbiome is closely linked to both vaginal and general health, and for this reason, microbiome studies of the vagina are an area of research. In this study, we analyzed the vaginal microbiome of women before and after menopause to further increase our understanding of the vaginal microbiome and its contribution to general health. We did a 16s rRNA gene-based metagenomic analysis on the vaginal fluids of 11 premenopausal and 19 postmenopausal women in Korea. We confirmed that the taxonomic composition was significantly different between the two groups. In postmenopausal women, species richness was significantly decreased, but species diversity was significantly increased. In particular, among the taxonomic components corresponding to all taxon ranks of the vaginal microbiome, a reduction in *Lactobacillus* taxa after menopause contributed the most to the difference between the two groups. In addition, we confirmed through metabolic analysis that the lactic-acid concentration was also decreased in the vaginal fluid of women after menopause. Our findings on the correlation between menopause and the microbiome could help diagnose menopause and enhance the prevention and treatment diseases related to menopause.

## Introduction

The “microbiome” encompasses all microorganisms and their genomes that inhabit humans, animals, plants, the natural environment, and their surrounding environmental conditions [1]. According to the Human Microbiome Project Consortium report, 4,180 kinds of microorganisms inhabit human feces, and 775, 857, 1,267, and 255 kinds of microorganisms inhabit the buccal mucosa, anterior nares, supragingival plaque, and posterior fornix, respectively [2]. About 38 trillion microorganisms inhabit the average body compared to about 30 trillion cells; therefore, the ratio of the two is close to unity [3]. With this new appreciation of its scale, the importance of the human microbiome is coming into greater focus.

The human microbiome is called our second genome or our other genome because it is closely related to disease and health [4, 5]. According to MetaHIT (the European Union Project on Metagenomics of the Human Intestinal Tract), the number of genes in the human body is 3,299,822, which is more than 100 times the approximately 23,000 genes that constitute the human genome [6]. These human microbiome genes are classified into 25 groups by functions such as energy metabolism and are connected through a network to perform metabolic functions [6]. For this reason, the human microbiome is associated with numerous diseases, including inflammatory bowel disease, multiple sclerosis, diabetes, allergies, asthma, autism, and cancer, which is why much recent research has been conducted in this regard [7].

Over the years, the female vaginal ecosystem has been formed by co-evolution between a specific microbial partner and a human host [8]. Vaginal secretions contain numerous microbes associated with various diseases, including sexually transmitted infections, bacterial vaginosis, fungal infections, and preterm birth [9], and recently have begun to be analyzed in detail [10]. More specifically, vaginal microbiota analyses of women of childbearing age [11], women with bacterial vaginosis [12], women with pelvic inflammatory disease [13], and of women during pregnancy and the postpartum period, have been conducted [14]. In addition, studies have shown that the microbial environment of the vagina differs from race to race, so studies on the vaginal microbiome need to be carried out with such distinctions in mind [15].

Women typically spend the last third of their lives in menopause following the end of their reproductive years [16]. During menopause, women experience changes in sex hormone levels and various aging-related symptoms [16]. Menopause is associated with vasomotor dysfunction, vaginal dryness, mood changes, sleep disturbances, urinary incontinence, cognitive changes, somatic complaints, sexual dysfunction, and poor quality of life, caused by decreased secretion of the ovarian hormones, estrogen and progesterone [17]. Low-dose estrogen therapy has been used for menopausal women, and although such hormone therapy is effective, it has limitations due to side effects, such as mastodynia, fluid retention, nausea, lower extremities cramps, and headaches [18]. Recently, microbiome research has been done on menopausal women to alleviate both diseases caused by menopause and the limitations of existing treatments [19-21].

In this study, we analyze the vaginal microbiome of pre-menopausal and post-menopausal Korean women, and investigate the causes of menopause from a microbiome perspective.

## Materials and Methods

### Subjects and Sample Collection

We enrolled 30 women in the study (18–70 years), including 11 pre-menopausal and 19 post-menopausal women (Table 1). None of the participants had used antibiotics or had had vaginal infections within one month of screening, had not used estrogen products systemically within six months of sampling, and had not used topical estrogen-containing products within one week of sampling. In addition, post-menopausal women had all had amenorrhea for more than 12 months. Women were excluded if they had used douches or lubricants within the prior week. After obtaining informed consent, each participant completed a questionnaire about vaginal or urinary tract symptoms and any history of infection. They self-assessed the severity of several vaginal symptoms, such as vaginal dryness, vaginal irritation or itchiness, pain during urination not associated with infection, vaginal soreness, pain during sexual intercourse, and bleeding after sexual intercourse. The evaluation criteria were classified into mild, moderate, or severe symptoms. After the patients were placed in a lithotomy position on a seismic bed, we inserted a disposable speculum (Taizhou Kangjian Medical Equipment Co., Ltd., China) to collect vaginal secretions using a sterile cotton swab kit and immediately placed the cotton swab into a bucket containing distilled water. After insertion, we closed the lid and moved the bucket to the laboratory. All samples were collected at room temperature, immediately placed in 1 ml of sterile normal saline, then sent to the laboratory within 10 min for DNA extraction. This study was approved by the Soonchunhyang University Cheonan Hospital Ethics Committee (eIRB) (IRB No. 2019-10-017-005).

### Preparation of 16S rRNA Gene Amplicon Libraries, Sequencing

A diluted saline solution of vaginal secretion was taken and placed in a Lysing Matrix B tube with 0.1-mm-diameter glass beads (MP Biomedicals, USA). We performed bead-beating using a FastPrep-24 5G instrument (MP Biomedicals) for 30 s. We amplified the V4 region of the 16S rRNA gene using primers containing overhang sequences compatible with the Illumina Nextera XT index. The forward primer sequence was 515F (5’-TCGTCGGCAGCGTCAGATGTGTATAAGAGACAG‐GTGCCAGCMGCCGCGGTAA-3’), and the reverse was 806R (5’ GTCTCGTGGGCTCGGAGATGTGTATAAGAGACAG-GGACTACHVGGGTWTCTAAT-3’). We carried out all PCR reactions using a 2×KAPA HiFi HotStart ReadyMix (Kapa Biosystems, USA). Reactions were run with the following cycling parameters: initial denaturation at 95°C for 3 min; 25 cycles of 95°C for 30 s; 55°C for 30 s; 72°C for 30 s; and a final extension at 72°C for 5 min. Subsequently, we performed PCR cleanup.

PCR plates were centrifuged at 1,000 g for one minute at 20°C to collect condensation. We vortexed AMPure XP beads (Beckman Coulter, UK) for 30 s and added 20 μl aliquots to each well of the PCR plate. The entire volume was gently pipetted up and down 10 times, followed by incubation at room temperature for 5 min. The PCR plate was placed on a magnetic stand for 2 min. With the amplicon PCR plate on the magnetic stand, we removed supernatants using a multichannel pipette and washed the beads twice with freshly prepared 80% ethanol. After allowing beads to air-dry for 10 min, we removed the amplicon PCR plate from the magnetic stand and added 52.5 μl of 10 mM Tris (pH 8.5) to each well. After mixing the wells, we incubated the plate at room temperature for 2 min and placed it on the magnetic stand for 2 min. We transferred 50 μl of the supernatant from the amplicon PCR plate to a new 96-well PCR plate.

Following the manufacturer’s protocol, the metagenomic library was prepared using a Nextera XT DNA Library Prep Kit (Illumina, USA). After we mixed 5 μl of DNA, Nextera XT Index Primer 1, Nextera XT Index Primer 2, 25 μl of 2×KAPA HiFi HotStart ReadyMix (KAPA Biosystems), and 10 μl of PCR Grade water by pipetting, we performed PCR using the following program: initial denaturation at 95°C for 30 s; 8 cycles of 95°C for 30 s; 55°C for 30 s, 72°C for 30 s; and a final extension step at 72°C for 5 min. Subsequently, we performed PCR cleanup again. The sample was finally diluted twice from 1 nM to 50 pM with 10 mM Tris (pH 8.5). After adding a 10% PhiX Control library (Illumina), we loaded the library onto an iSeq-100 reagent cartridge (Illumina) and sequenced it on an iSeq-100 (Illumina).

### Metagenomics Data Analysis and Statistical Analysis

The FASTQ files obtained by sequencing were subjected to data analysis on the EzBioCloud server (Chunlab, Korea). The raw reads were then pre-processed using Trimmomatic [22] to trim adapters and low-quality bases to produce clean reads. The primers were then trimmed with Myers & Miller's alignment algorithm [23] at a similarity cutoff of 0.8. Non-specific amplicons that do not encode 16S rRNA were detected by nhmmer in the HMMER software package with hmm profiles [24]. Unique reads were extracted, and redundant reads were clustered with VSEARCH_2_'s derep_fulllength command [25]. EzBioCloud 16S database [26] was used for a taxonomic assignment using VSEARCH's userarch_global command [25], followed by more precise pairwise alignment in Myers & Miller's algorithm [23].

Alpha (α) diversity indices were analyzed based on ACE (abundance-based coverage estimator) [27], Chao1 [28], Jackknife [29], Shannon/Simpson [30], NPShannon (non-parametric Shannon) [31], and Phylogenetic diversity [32], and beta (β) diversity indices were analyzed based on Jenson-Shannon [33], Bray-Curtis [34], Generalized UniFrac [35], and Fast UniFrac [36]. Taxonomic and functional biomarkers were discovered by statistical comparison algorithms of the linear discriminant analysis (LDA) effect size (LEfSe) [37] and Kruskal-Wallis H tests [38].

### Assessment of Organic Acids in Vaginal Samples

Vaginal swabs were centrifuged at 14,000 g for 10 min in a cold room (4–8°C). Supernatants were transferred to new 1.5 ml microcentrifuge tubes, and methanol (cooled to −80°C) was added to make a final 80% (v/v) methanol solution. We gently shook the solution to mix it and incubated it for 6–8 h at −80°C. Then, it was centrifuged at 14,000 g for 10 min (4–8°C). The supernatant was again transferred to new 1.5 ml microcentrifuge tubes and glass vials for X500B QTOF [39]. Briefly, we performed LC-MS analysis using Exion LC AD coupled with the X500B QTOF system (AB Sciex Pte. Ltd., USA). We injected samples into an ACQUITY UPLC BEH HILIC column (2.1 × 50 mm, 1.7 μm, Waters, USA). The mobile phase comprised phase A (water with 10 mM ammonium formate) and phase B (methanol). We performed auto-calibration on every fifth sample. We adapted SCIEX OS software 1.0 to analyze data.

### Culture of *Lactobacillus* from Vaginal Fluid

The collected vaginal fluid samples were stored at -80°C with 15% glycerol and used for CFU analysis later. To confirm the *Lactobacillus* contained in the vaginal fluid of a woman, a culture method using MRS (de Man, Rogosa, and Sharpe) media was performed as in the previous study [40]. On Difco *Lactobacillus* MRS Agar (288130, BD, USA) plates, 100 μl of the collected vaginal sample was spread using a spreader. The plates were then incubated at 37°C for 3 days. After incubation, the number of colonies was analyzed.

## Results

### Microbial Diversity in Vaginal Metagenomes of Pre-menopausal and Post-menopausal Women

**Fig. 1:**
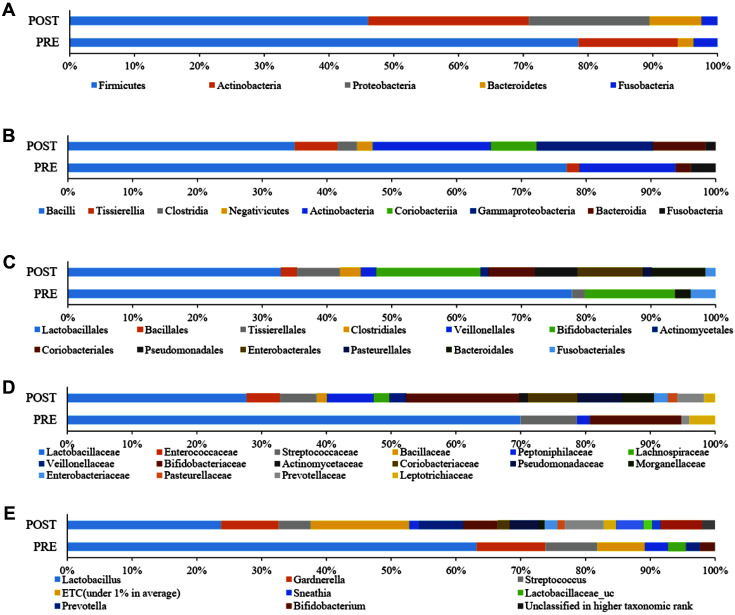
Average taxonomic compositions of pre-menopausal and post-menopausal women. The pre-menopause group (PRE) and post-menopause group (POST) were further classified at the (**A**) phylum, (**B**) class, (**C**) order, (**D**) family, and (**E**) genus. Those with relative abundances less than 1% were expressed as ETC.

Table 2 and Figs. 1A-1D shows taxonomic composition at the level of phylum, class, order and family for pre-and post-menopausal women. Analyzing taxonomic classification at the phylum level, it was found that Firmicutes accounted for the highest percentage, followed by Actinobacteria, Proteobacteria, Bacteroides, and Fusobacteria. The proportions for each of the phyla were 77.8%, 15.2%, 0.0%, 2.4%, and 3.7% in pre-menopausal women, respectively, and 46.1%, 24.9%, 18.6%, 8.0%, and 2.5% in post-menopausal women, respectively. Firmicutes was significantly reduced (*p* = 0.012), but Proteobacteria (*p* = 0.0004) and *Bacteroidetes* (*p* = 0.019) were significantly increased in post-menopausal compared to pre-menopausal women. At the class level, Bacilli, which correspond to the Firmicutes phylum, accounted for the highest proportion and were significantly decreased (*p* = 0.008) in post-menopausal women (34.3%) compared to pre-menopausal women (74.7%). In contrast, Tissierellia, Clostridia, Negativicutes, Coriobacteriia, and Gammaproteobacteria were significantly increased post-menopausally compared to pre-menopausal (*p* = 0.005, 0.013, 0.023, 0.006, and 0.002, respectively). At a lower taxonomic level, Lactobaciilales order was significantly depleted (*p* = 0.009) in post-menopausal (31.8%) with respect to pre-menopausal women (74.3%). On the other hand, Bacillales (*p* = 0.021), Tissierellales (*p* = 0.005), Clostiridiales (*p* = 0.013), Veillonellales (*p* = 0.023), Actinomycetales (*p* = 0.012), Coriobacteriales (*p* = 0.006), Pseudomonadales (*p* = 0.012), and Enterobacterales (*p* = 0.004) were significantly increased post-menopausally. Analyzing the vaginal microbiota composition at the family level, *Lactobacillaceae* was significantly decreased (*p* = 0.033) post-menopausally (24.7% compared to 65.9%); whereas *Peptoniphilaceae* (*p* = 0.005), *Veillonellaceae* (*p* = 0.023), *Actinomycetaceae* (*p* = 0.012), *Coriobacteriaceae* (*p* = 0.006), *Pseudomonadaceae* (*p* = 0.03), *Morganellaceae* (*p* = 0.008), *Enterobacteriaceae* (*p* = 0.016), and *Prevotellaceae* (*p* = 0.045) showed significant increases in post-menopausal women. At the genus level, *Lactobacillus* showed significant reductions (*p* = 0.037) post-menopausally (23.7% compared to 63.2%), whereas *Prevotella* (*p* = 0.023), unclassified *Lactobacillaceae* (*p* = 0.033), *Escherichia* (*p* = 0.041), *Pseudomonas* (*p* = 0.03), *Proteus* (*p* = 0.017), *Finegoldia* (*p* = 0.006), and *Atopobium* (*p* = 0.006) were significantly increased after menopause (Fig. 1E).

### Correlation between Pre-Menopause and Post-menopause Groups

**Fig. 2:**
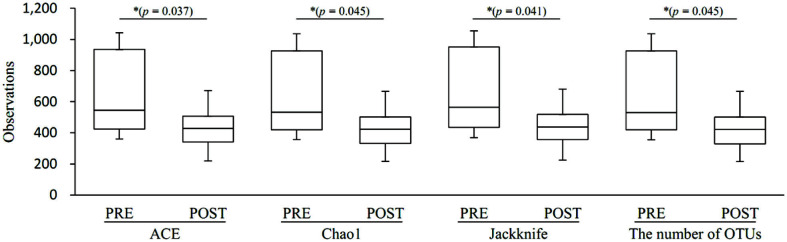
Boxplot of species richness indices. The species richness of the pre-menopause group (PRE) or post-menopause group (POST) was analyzed by Ace, Chao1, Jackknife, and the number of OTUs method. The horizontal thick black band represents the median value, and the boxplot margins indicate the first and third quartiles.

**Fig. 3:**
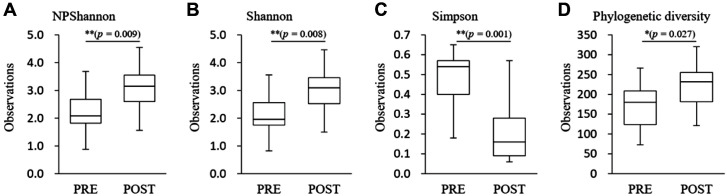
Boxplot of species diversity indices. The species diversity of the pre-menopause group (PRE) and post-menopause group (POST) was analyzed by (**A**) NPShannon, (**B**) Shannon, (**C**) Simpson (*p* = 0.007), and (**D**) phylogenetic diversity. The horizontal thick black band represents the median value, and the boxplot margins indicate the first and third quartiles.

**Fig. 4:**
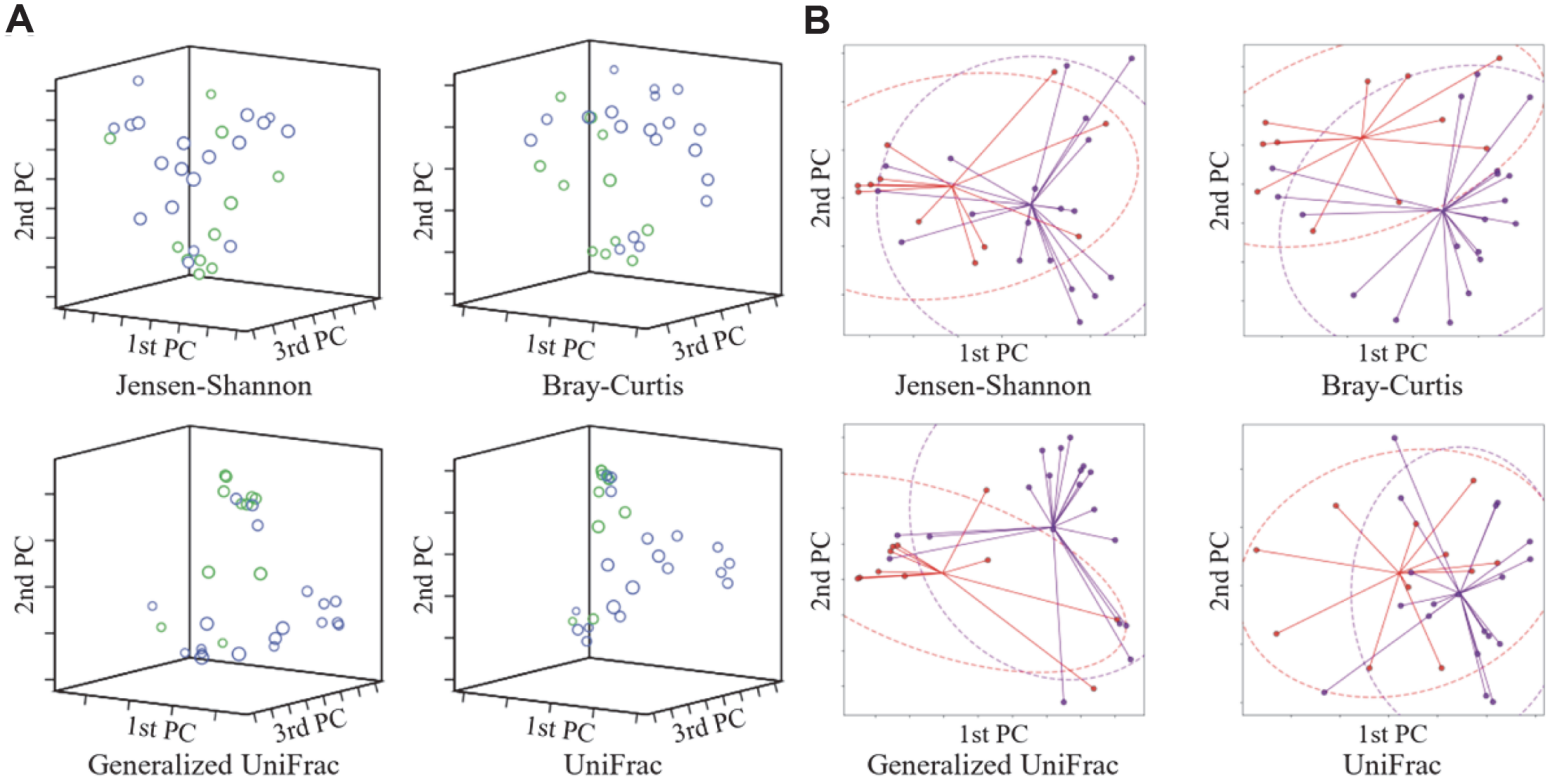
Principal coordinate analysis (PCoA) of the bacterial communities present in the pre-menopause group (PRE) and post-menopause group (POST). (**A**) Beta diversity 3D PCoA plot based on Jensen-Shannon divergence, Bray-Curtis, Generalized UniFrac, and UniFrac. (**B**) Beta diversity PCoA plot with an ellipse at 95% confidence interval based on Jensen-Shannon divergence, Bray-Curtis, Generalized UniFrac, and UniFrac. The blue/purple color indicates post-menopausal, and the light green/red color indicates pre-menopausal, respectively.

**Fig. 5:**
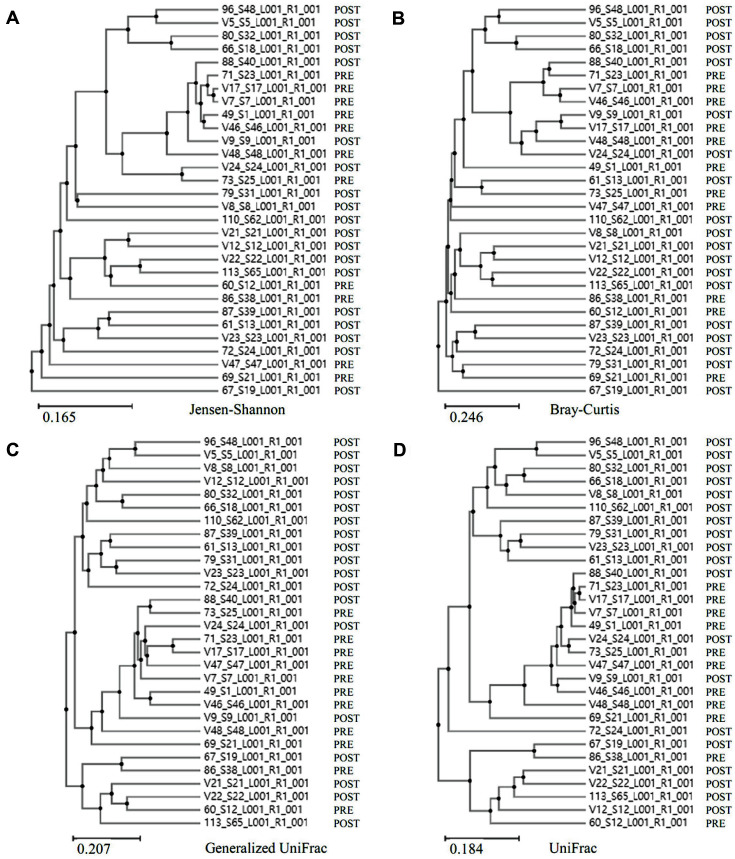
Clustering using the Unweighted Pair Group Method with Arithmetic mean (UPGMA). Pre-menopause group (PRE) and post-menopause group (POST) were analyzed by (**A**) Jensen-Shannon, (**B**) Bray-Curtis, (**C**) Generalized UniFrac, and (**D**) UniFrac.

Species richness in the pre-menopause group was significantly higher than in post-menopause (Ace, *p* = 0.037; Chao1. *p* = 0.045; Jackknife, *p* = 0.041; the number of OTUs, *p* = 0.045) (Fig. 2). In species diversity, the Simpson index was significantly lower (Simpson, *p* = 0.001) and was significantly higher in the NPShannon, Shannon, and phylogenetic diversity indices (NPShannon, *p* = 0.009; Shannon, *p* = 0.008; phylogenetic diversity, *p* = 0.027) in the post-menopause samples (Fig. 3). Principal coordinate analysis (PCoA) plots revealed significant differences between the pre-menopausal and post-menopausal groups based on the Unweighted Pair Group Method using Jensen-Shannon divergence, Bray-Curtis, Generalized UniFrac, and UniFrac (Fig. 4). Unweighted Pair Group Method with Arithmetic mean (UPGMA) hierarchical clustering was analyzed in Fig. 5. Beta-set-significance analysis showed significant differences in the genus or species level between the pre-menopausal and post-menopausal groups (Table 3).

### Taxonomic Biomarker Discovery

**Fig. 6:**
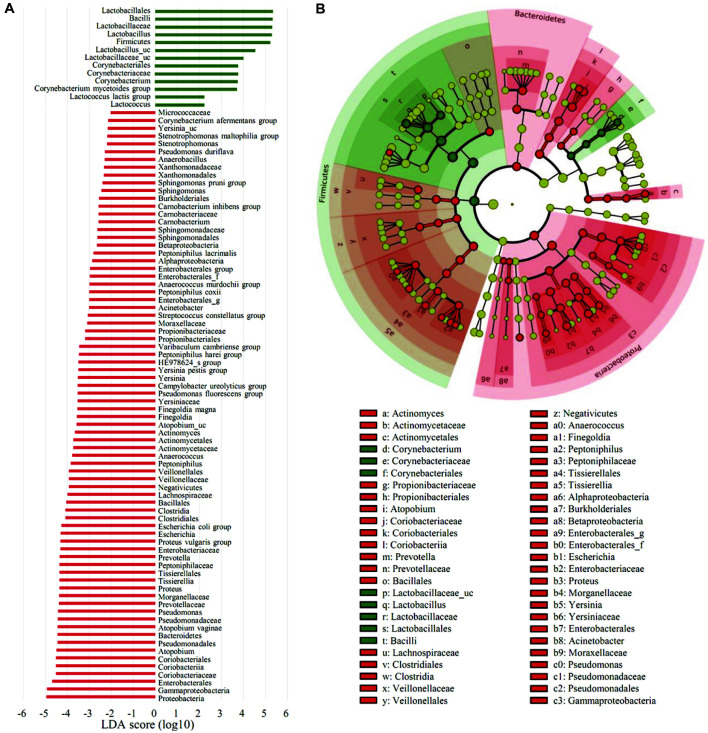
Distinct taxa identified in the pre-menopause group (PRE) and post-menopause group (POST) using LEfSe (Linear discriminant analysis Effect Size) analysis. (**A**) Taxonomic cladograms were derived from a LEfSe analysis with linear discriminant analysis (LDA) scores greater than 2 and significance at α < 0.05, as determined by the Kruskal-Wallis test. (**B**) Cladogram representing statistically significant differences in bacterial clades between premenopausal and post-menopausal women. Small circles shaded with different colors in the diagram represent the abundances of the taxa in each respective group. Each circle's diameter is proportional to the taxon's abundance. Regions in red indicate taxa enriched in the post-menopause group, while regions in green indicate taxa enriched in the pre-menopause group.

**Fig. 7:**
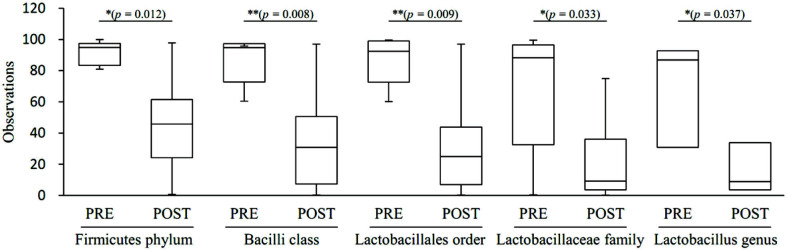
Taxonomic abundance with an LDA effect size of more than 5. Taxonomic relative abundance was analyzed for the two groups using boxplots. The horizontal thick black band represents the median value, and the boxplot margins indicate the first and third quartiles. Statistical significance between groups was analyzed using the Wilcoxon rank-sum test (*, *p* < 0.05; **, *p* < 0.01).

To identify the specific bacterial taxa associated with menopause, we compared the vaginal microbiota groups using LEfSe in the Galaxy workflow framework with the parameters set at *p* < 0.05 and LDA score = 2.0 (Fig. 6A). Only those with *p* < 0.05 and LDA effect size > 4 are summarized in Table 4. Taxonomic cladogram obtained from LEfSe analysis of 16S sequences is shown in Fig. 6B. The taxa with an LDA effect size exceeding 5 included Lactobacillales (5.34), Bacilli (5.32), *Lactobacillaceae* (5.31), *Lactobacillus* (5.29), and Firmicutes (5.23) (Fig. 7).

### Vaginal Organic Acids by Metabolomics Analysis

**Fig. 8:**
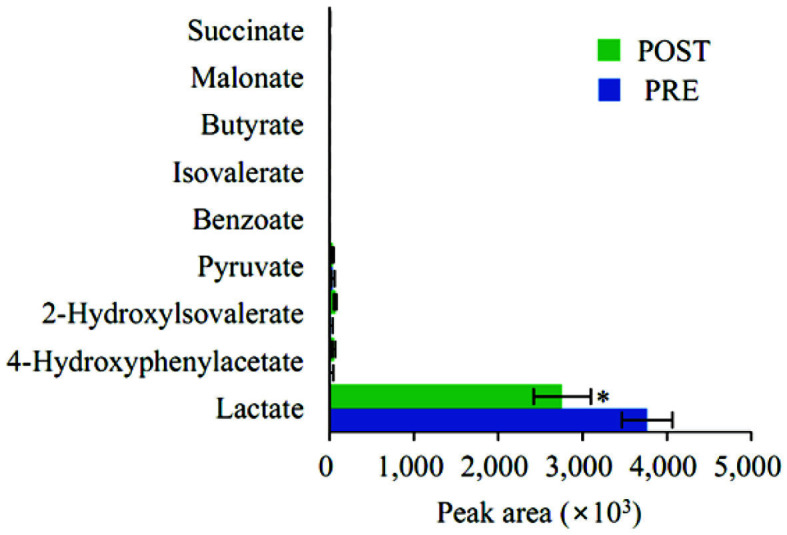
The relative levels of organic acids in pre- and post-menopausal vaginal secretions. Data are presented as means with standard errors. *, *p* < 0.05; PRE, pre-menopause group; POST, post-menopause group.

Fig. 8 shows the results of a quantitative analysis of organic acids in vaginal fluid. In the pre-menopausal group, lactate accounted for the highest percentage, 98.0% of the total organic-acid content. Post-menopausally, lactate concentration was 94.2%, which was significantly lower (*p* = 0.015). The concentrations of pyruvate, 4-hydroxyphenyl acetate, 2-hydroxylovalerate, succinate, benzoate, isovalerate, butyrate, and malonate were lower than that of lactate, and there were no significant differences for any of these.

### Quantification of *Lactobacillus* from Vaginal Fluid

**Fig. 9:**
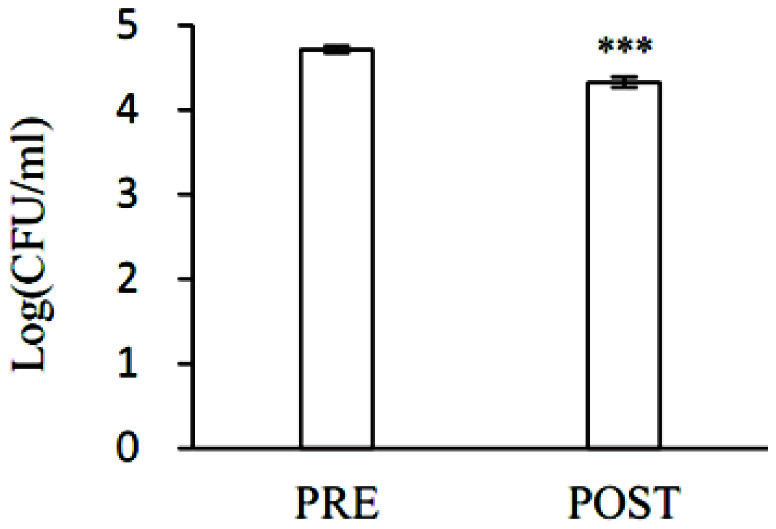
Quantitative comparison of live *Lactobacillus* in vaginal fluid. Y-axis means CFU of *Lactobacillus* in the collected vaginal fluid. Values are expressed as mean and standard deviations. Statistical significance of the post-menopausal (POST) group compared to the pre-menopausal (PRE) group was analyzed using an unpaired Student's *t*-test. ***, *p* < 0.001.

*Lactobacillus* in vaginal fluid samples collected was quantitatively analyzed through the culture method using MRS media (Fig. 9). *Lactobacillus* in vaginal fluid samples was 5.1 × 10^4^ CFU/ml in pre-menopausal women and 2.1×10^4^ CFU/ml in post-menopausal women. The amount of *Lactobacillus* in post-menopausal women confirmed by the culture method was 41.1% of the amount of *Lactobacillus* in pre-menopausal women, which was statistically significantly lower (***p < 0.001).

## Discussion

About 20 years ago, the human microbiome was a fledgling field, but it is now thriving with investigations integrating basic and clinical sciences [41]. Microbiome studies of the digestive system have found associations between the human gut microbiome and cancer, neurodegenerative diseases, obesity, diabetes, and liver diseases; and by extension, the human gut microbiota is considered a potential source of new therapeutic agents [42]. Vaginal microbiome research has also been conducted over the past 10 years and has been found to be essential for maintaining vaginal health and host protection from disease and is related to behavior, health outcomes, race, ethnicity, and hygiene [43].

In this study, we performed a vaginal microbiome analysis on 11 pre-menopausal women and 19 post-menopausal women. There was a statistically significant difference in the composition of the taxonomic distribution in the two groups. *Lactobacillus* taxa dominated in pre-menopausal women, but our data confirmed that it decreased after menopause. In this survey, the *Lactobacillus* genus decreased from 63.2% pre-menopausal to 23.7% post-menopausal. This result is consistent with an earlier study in American women, which showed a reduction in the *Lactobacillus* genus from 83 to 54% post-menopause [44]. However, quantitatively, there was a difference between the two studies, and it seems that there is a need for studies in different ethnic groups.

Species abundance was lower, and species diversity was higher in post-menopausal women than in pre-menopausal women. This difference is probably due to the reduced abundance of *Lactobacillus*, which acts as an antimicrobial, and as a result, pathogens that previously colonized the vagina at a low rate have multiplied or become newly infectious. In a vaginal microbiome analysis of a female patient with pelvicitis in China, a decrease in lactobacilli was accompanied by polymicrobial infection, consistent with the current results [45].

LEfSe analysis showed that among all taxa corresponding to all taxon ranks, the decrease in the *Lactobacillus* taxa had the most considerable effect on the differences seen before and after menopause. In addition, metabolite analysis confirmed that lactic acid, produced mainly by *Lactobacillus*, was present at significantly lower concentrations in post-menopausal women. *Lactobacillus* can cause vaginal eubiosis by killing dysbiotic microbes and various pathogens through lactic acid production, a primary antibacterial substance [46]. From these results, we would predict that *Lactobacillus* may be used to alleviate diseases related to menopause. A clinical trial in Korea reported that oral ingestion of lactobacilli by post-menopausal women improved symptoms related to menopause [47], but further studies are needed to investigate alleviation of menopause-related symptoms by administering *Lactobacillus* directly to the vagina rather than by oral administration to restore the vaginal microbiome to a pre-menopausal state.

In addition to menopause, the vaginal microbiome is associated with vaginal infections, such as pelvic vaginitis, bacterial vaginosis, vulvar candidiasis, sexually transmitted diseases, and human immunodeficiency virus (HIV) infection [48, 49]. Also, changes in the vaginal microflora can lead to severe gynecological problems, such as pregnancy loss, premature birth, and low pregnancy rates [50]. Therefore, the results of this study will contribute to broadening the understanding of not only menopause but also various gynecological diseases, and further studies will be needed for this. However, it is considered as a limitation of this study that the study subjects are not sufficient. In addition, more in-depth studies and analyses of the microbiome according to menopause by age group are needed.

## Figures and Tables

**Table 1:** Clinical profiles of Korean pre-menopausal (*n* = 11) and post-menopausal women (*n* = 19).

Characteristics	Pre-menopausal (*n* = 11 )	Post-menopausal (*n* = 19 )
Age (years) (mean±SD)	39.4±2.4	58.5 ± 3.6
BMI (mean±SD)	23.2±3.3	25.9 ± 3.6

SD, standard deviation; BMI, Body mass index

**Table 2:** Distributions of bacterial community structures at different taxonomic levels (phylum, class, order, and family).

Phylum	PRE	POST	*p*-value	Class	PRE	POST	*p*-value	Order	PRE	POST	*p*-value	Family	PRE	POST	*p*-value
Firmicutes	77.8	46.1	0.012*	Bacilli	74.7	34.3	0.008**	Lactobacillales	74.3	31.8	0.009**	Lactobacillaceae	65.9	24.7	0.033*
												Enterococcaceae	0.0	4.6	0.846
												Streptococcaceae	8.2	5.1	0.107
								Bacillales	0.0	2.4	0.021*	Bacillaceae	0.0	1.4	0.22
				Tissierellia	1.9	6.5	0.005**	Tissierellales	1.9	6.5	0.005**	Peptoniphilaceae	1.9	6.5	0.005**
				Clostridia	0.0	3.0	0.013*	Clostridiales	0.0	3.0	0.013*	Lachnospiraceae	0.0	2.1	0.037*
				Negativicutes	0.0	2.3	0.023*	Veillonellales	0.0	2.3	0.023*	Veillonellaceae	0.0	2.3	0.023*
Actinobacteria	15.2	24.9	0.067	Actinobacteria	14.4	17.9		Bifidobacteriales	13.3	15.6	0.116	Bifidobacteriaceae	13.3	15.6	0.116
								Actinomycetales	0.0	1.2	0.012*	Actinomycetaceae	0.0	1.2	0.012*
				Coriobacteriia	0.0	6.9	0.006**	Coriobacteriales	0.0	6.9	0.006**	Coriobacteriaceae	0.0	6.9	0.006**
Proteobacteria	0.0	18.6	0.0004**	Gammaproteobacteria	0.0	17.6	0.002**	Pseudomonadales	0.0	6.4	0.012*	Pseudomonadaceae	0.0	6.1	0.03*
								Enterobacterales	0.0	9.8	0.004**	Morganellaceae	0.0	4.4	0.008**
												Enterobacteriaceae	0.0	1.9	0.016*
								Pasteurellales	0.0	1.3	0.651	Pasteurellaceae	0.0	1.3	0.651
Bacteroidetes	2.4	8.0	0.019*	Bacteroidia	2.3	8.0	0.05	Bacteroidales	2.3	8.0	0.05	Prevotellaceae	1.2	3.7	0.045*
Fusobacteria	3.7	2.5	0.272	Fusobacteria	3.7	1.5	0.272	Fusobacteriales	3.7	1.5	0.272	Leptotrichiaceae	3.7	1.5	0.292

Unit: %; Those present at less than 1% are not included. *p < 0.05; **p < 0.01.

PRE, pre-menopause group; POST, post-menopause group.

**Table 3:** Statistical analysis of beta-diversity.

Beta diversity distance	Species	Genus
Jensen-Shannon	*(*p* = 0.018)	*(*p* = 0.029)
Bray-Curtis	*(*p* = 0.011)	**(*p* = 0.008)
Generalized UniFrac	**(*p* = 0.002)	**(*p* = 0.002)
UniFrac	** (*p* = 0.004)	** (*p* = 0.004)

Permutational multivariate analysis of variance (PERMANOVA) results demonstrated the beta set significance between premenopausal and post-menopausal women. *p < 0.05; ** *p* < 0.01.

**Table 4:** Kruskal-Wallis H tests and LEfSe analysis identification of associations between pre-menopausal and post-menopausal women.

Taxon name	Taxon rank	LDA effect size	*p*-value	PRE (%)	POST (%)
Lactobacillales	Order	5.34	0.01	74.34	31.84
Bacilli	Class	5.32	0.01	74.68	34.27
Lactobacillaceae	Family	5.31	0.03	65.87	24.69
Lactobacillus	Genus	5.29	0.04	63.16	23.73
Firmicutes	Phylum	5.23	0.01	77.84	46.10
Proteobacteria	Phylum	4.94	0.00	0.82	18.65
Gammaproteobacteria	Class	4.92	0.00	0.76	17.64
Enterobacterales	Order	4.68	0.00	0.42	9.82
Lactobacillus_uc	Species	4.54	0.03	9.72	2.62
Coriobacteriales	Order	4.51	0.01	0.76	6.94
Coriobacteriia	Class	4.51	0.01	0.76	6.94
Coriobacteriaceae	Family	4.51	0.01	0.76	6.94
Atopobium	Genus	4.50	0.01	0.45	6.43
Pseudomonadales	Order	4.45	0.01	0.31	6.38
Bacteroidetes	Phylum	4.44	0.02	2.36	8.03
Atopobium vaginae	Species	4.44	0.01	0.27	5.50
Pseudomonadaceae	Family	4.43	0.03	0.29	6.13
Pseudomonas	Genus	4.42	0.03	0.29	6.00
Prevotellaceae	Family	4.38	0.05	2.24	7.31
Morganellaceae	Family	4.37	0.00	0.00	4.38
Proteus	Genus	4.36	0.01	0.00	4.26
Tissierellia	Class	4.36	0.00	1.92	6.54
Tissierellales	Order	4.36	0.00	1.92	6.54
Peptoniphilaceae	Family	4.36	0.00	1.92	6.54
Prevotella	Genus	4.34	0.05	2.22	6.87
Enterobacteriaceae	Family	4.31	0.02	0.38	4.58
Proteus vulgaris group	Species	4.31	0.01	0.00	3.83
Escherichia	Genus	4.30	0.04	0.37	4.43
Escherichia coli group	Species	4.26	0.04	0.33	4.03
Clostridia	Class	4.08	0.01	0.46	2.98
Clostridiales	Order	4.08	0.01	0.46	2.98
Bacillales	Order	4.04	0.02	0.29	2.42
Lactobacillaceae_uc	Genus	4.00	0.02	2.70	0.71

Only those with *p* < 0.05 and linear discriminant analysis (LDA) effect size > 4 are presented.

% refers to the percentage of distribution occupied by each group.

PRE, pre-menopause group; POST, post-menopause group.
